# Dyad learning versus individual learning under medical simulation conditions: a systematic review

**DOI:** 10.12688/mep.19285.1

**Published:** 2022-11-28

**Authors:** Jack Ding, Xin Xiao, Shanon Biagi, Thomas Varkey

**Affiliations:** 1 Faculty of Health and Medical Sciences, University of Adelaide, Adelaide, South Australia, 5000, Australia; 2 Department of Medical Education, Melbourne Medical School, Melbourne, Victoria, 3010, Australia; 3 California University of Science and Medicine, Colton, California, USA; 4 Department of Medical Education, Dell Medical School, University of Texas at Austin, Austin, Texas, USA; 5 The College of Medicine, The University of Arizona, Phoenix, Arizona, USA

**Keywords:** dyad, medical simulation, medical education, dyadic learning

## Abstract

**Background: **Dyad learning is a two-person learning dynamic in which one student observes the other performing tasks, with their roles then being reversed such that both students experience the observer and the performer role. The efficacy of dyad learning has been tested in medical education contexts, such as in medical simulation. To our knowledge, this is the first systematic review that has evaluated the efficacy of dyad learning in a medical simulation context.

**Methods: **PubMed, Google Scholar, Cochrane Library databases were searched in September 2021 and January 2022. Prospective studies of randomized design that compared dyad learning to a single medical student or physician learning in a medical simulation were included. Non-English language studies, secondary literature papers, non-human based studies, and papers that were published prior to 2000 were excluded. The methodological quality of these studies was assessed using the Medical Education Research Study Quality Instrument (MERSQI). The Kirkpatrick model was used to conceptualize study outcomes.

**Results:** The identified papers included eight studies from four countries that totaled 475 participants. Students reported positively on their experiences as dyads, especially regarding the social aspects of it. Studies showed non-inferior learning outcomes for dyads. As most studies were one or two days long, there is limited evidence that this non-inferiority extends to longer term training modules. There is some evidence to suggest that dyad learning outcomes may be replicable in a clinical context following simulation training.

**Conclusions:** Dyad learning in medical simulation is a pleasant experience for students and may be as effective as conventional learning. These findings set the foundation for future studies of longer duration, which is needed to determine the efficacy of dyad learning in lengthier curriculums and long-term knowledge retention. While cost-reduction is an implied benefit, studies that explicate cost reduction are needed to formalize this.

## Introduction

Over the last 30 years, simulation teaching has been adopted and integrated into pre-clinical and clinical curricula by medical educators internationally ^1^. Medical simulation enables student exposure to challenging and stressful situations in standardized and controlled environments, without conveying any true physical risk to patients ^2^.

However, with the expansion of medical simulation, there is an increased pressure to justify associated expenditures, in both higher and lower-income countries ^3^. For instance, a 2011 American paper reported on the economic aspects of employing a 4-week simulation training program for 38 surgical residents at a single institution. The total implementation cost for the facility was calculated to be $4.2 million, with annual operating expenses totaling $476,000. In other words, the yearly investment for each trainee was $12,516 ^4^. If the ratio of learners to simulation equipment is increased 2:1, then expenditures can potentially be reduced, with more efficient allocation of funds or investment in a higher quality simulation environment. In addition to justifying costs, medical educators have an ongoing duty to optimize teaching efficacy and to maximize student wellbeing, and therefore need to be adaptable to strategies that may improve these elements ^5^. Prolonged periods of independent learning may incur a sacrifice of social facets of training and exposure to regular feedback and supervision. Dyadic learning may offset these disadvantages, by means of steady companionship, supervision, and feedback from a peer-learner ^2^.

Dyad learning is a training dynamic whereby two individuals collaboratively learn and apply a new skill, with the intention of ultimately fostering the ability to do so independent of one another ^6^. It is underpinned by multiple neurobiological mechanisms and theories, including concepts related to observational learning, self-regulated learning, cognitive load reduction, and meta-cognition.

There are many primary papers that explore the dyad training as an alternative to single student learning under simulation conditions. However, to our knowledge, a systematic collation and scrutinization of existing literature in this area does not exist. Therefore, the authors present this systematic review to bridge this gap in the medical education literature, and to recommend possible points for the future.

## Methods

This systematic review followed the Preferred Reporting Items for Systematic Review and Meta-Analysis (PRISMA) 2020 statements ^7^. The review protocol was not prospectively registered. The template data collection forms and other materials used are available on request.

### Search strategy

A literature search was conducted by two authors (JD and TV) in September 2021, using the databases of PubMed, Google scholar, and Cochrane Central Register of Controlled Trials (CENTRAL). This search was repeated by a third author (SB) in January 2022. The term “dyad” was searched in PubMed and Google Scholar in combination with each of the following terms: medical student, learning, practice, training, and simulation. The precise search phrase used in PubMed and Google Scholar is detailed as follows, noting the Boolean operator, quotation marks, and parentheses: “DYAD” AND (medical student OR learning OR practice OR training OR simulation). The term “dyad” was used in isolation for searching the Cochrane Central Register.

### Selection strategy

Reviewers independently assessed the titles of papers discovered from the abovementioned search strategy for potential relevance. Following completion of this search process, duplicate papers within the cumulative study pool were then removed. The remaining papers were then screened for relevance based on the abstract. The full texts of the articles that were deemed possibly relevant based on the abstract were retrieved and assessed for eligibility. If the paper did not meet our inclusion criteria or if they fulfilled the exclusion criteria, they were eliminated from further review. Disagreement at any stage of this process between the reviewers regarding relevance or use potential was resolved through discussion. 

The type of studies included for this review were those of prospective and randomized design, regardless of publication status (published journal articles, conference papers). The following inclusion and exclusion criteria were determined prior to the search process detailed in the prior paragraph.

Inclusion criteria

1. Presence of a control group
2. Presence of a dyad group
3. Participants were medical students or physicians
4. The outcome measured is relevant to medicine
5. A skill must be learned under a simulation setting

Exclusion criteria

1. non-English language publications (full text)
2. Secondary literature papers (reviews, commentaries, editorials, opinion pieces)
3. Studies published prior to 2000 (irrelevant due to the digital advancements of modern days)
4. Animal studies

‘Medical students’ was defined as students enrolled in an undergraduate or graduate medical program that culminates with the awarding of a medical degree recognized by the World Directory of Medical Schools. ‘Dyad’ was defined as a group of two individuals.

### Data extraction

Pertinent data from each of the selected full-text articles was independently extracted by two authors who used a standardized data collection form. Gathered information included authors, country, study design details, baseline competency of participants, teaching time, mode of teaching, skill acquired, method of evaluating skill acquisition, and outcome measures. Discrepancies were resolved by discussion between the two authors. If there were any unresolved disputes, a senior medical education faculty member of either medical school the authors were affiliated with would have been consulted.

### Data synthesis

An adapted variation of the Kirkpatrick model was used to conceptualize outcome measures ^8^. The Kirkpatrick model is a four-tiered framework for evaluating learning. It is frequently used to evaluate educational outcomes of healthcare training programs ^9^. The primary characteristics of each study in this paper were tabulated and compared against each other for synthesis at each tier. The four levels of the adapted Kirkpatrick model in ascending order include: reaction (individual responses and reactions to learning as dyads), learning (progression of learning as discerned through objective evaluation during or within a week of the teaching phases), behavior (independent application of the learned information at least one week after the teaching phase), and results (independent application of learned information under live, non-simulation conditions) ^10^.

### Quality assessment

The Medical Education Research Quality Instrument tool (MERSQI) is a well validated framework that is frequently used to evaluate the quality of education research ^11^. The methodological quality of each included study was independently appraised by two authors (LX, TV) using the MERSQI tool. Discrepancies in the results was resolved through discussion with a third author (JD).

## Results

**Figure 1.**
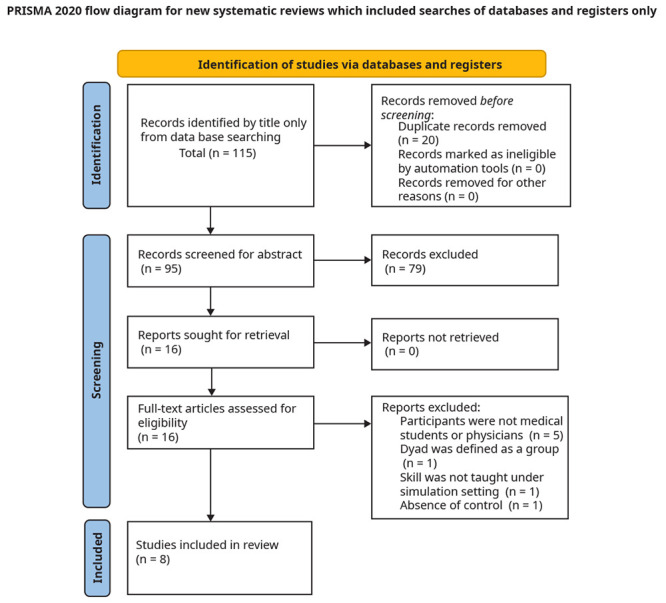
PRISMA flow diagram depicting the selection strategy for this systematic review.

The process of study selection is depicted in the below PRISMA 2020 flow diagram ( Figure 1).

This systematic review consists of eight studies. Most of the studies were conducted in Denmark ^12– 16^, one was in Germany ^17^, one was in Canada ^18^, and one was in the United States ^2^. Six studies were of two-arm randomized controlled trial (RCT) design ^2, 12– 15, 18^, one was a three-arm RCT ^17^, and one study was mixed-method RCT ^16^. The timeframes of the training programs of all but one study ^16^ was limited to no more than two days. In terms of content, two studies involved simulated patient encounters ^2, 14^, two studies trained surgical skills ^16, 17^, three studies taught medically invasive, non-surgical procedures ^12, 13, 18^, and one study trained ultrasonography skills ^15^. Only one study used a live follow-up to evaluate performance ^15^. The remaining studies conducted a follow-up assessment under simulation conditions ^2, 12– 14, 16– 18^.

Table 1 depicts the key characteristics of the studies analyzed in this systematic review.

**Table 1.** Pertinent characteristics of selected studies evaluating dyad learning and conventional learning outcomes in simulation conditions.

Authors	Country	Study design	Sample (dyad / single)	Skill learned	Teaching method	Evaluation method	General conclusions
Bjerrum, 2014 ^12^	Denmark	RCT	18/18, total n = 36 medical students	Bronchoscopy (novice level)	Supervised simulation training (video guide, 10 simulated cases, 20 minutes each in 1x 0.5-day session)	Objective simulator measures with 3-week follow-up	No significant group differences. Notably decreased dyad retention test results in two measures was not significantly different to control (p=0.27 and p = 0.20).
Kowalewski, 2019 ^17^	Germany	RCT	Three arm: dyad (n=40), single (n=40), no training (n=20), total n=100 medical students	Laparoscopic cholecystectomy (novice level)	Self-directed simulation training (2x 4-hour sessions)	Simulated (porcine) LC, blinded evaluators, OSATS, GOALS, time for LC, VR result	No significant group differences (between all 3 groups), for OSATS, GOALS. No significant difference in any VR reported parameters between dyad and single.
Räder, 2014 ^13^	Denmark	RCT	36/36, total n =72 medical students	Coronary angiography (novice level)	Self-directed simulation training (1x 3.5-hour session)	Simulated coronary angiography, blinded evaluators, MOCARS scale	No significant group differences in MOCARS scores. Single: 68% (SD 13%). Dyad: 63% (SD 16%). (p = 0.18).
Shanks, 2013 ^18^	Canada	RCT	26/24, total n = 50 resident physicians (17/11 at 2 months test, retention: 62%).	Lumbar puncture (novice level)	Self-directed simulation training with some supervisor input (1x practice simulation test and 1x 0.5-hour session)	Simulated lumbar puncture, blinded evaluators, GRS scoring	No significant group differences on GRS pre-test, post-test, retention- test scores
Tolsgaard, 2013 ^14^	Denmark	RCT	24/25, total n = 49 medical students	Patient encounter (history and physical exam at a pre- clinical level)	Supervised simulation training of clinical skills (1x 4-hour session, 4 scenarios).	Simulated patient encounter, blinded evaluators, scoring based on RIME framework	Dyad total score significantly higher than single group. Dyad: 40.7% (SD 6.6). Single: 36.9% (SD 5.8). (p = 0.04, effect size 0.61)
Tolsgaard, 2015 ^15^	Denmark	RCT	16/14, total n = 30 medical students	Transvaginal ultrasound (novice level)	Self-directed simulation training (1x 2-hour session)	Live transvaginal ultrasound, blinded evaluators, OSAUS scoring	No significant group differences in live OSAUS scores. Dyad: 56.3% (SD 14.8). Single: 48.4% (SD 11.8). (delta score 7.8%, 95% CI -3.8-19.6%).
Zetner, 2021 ^16^	Denmark	RCT	45/52, total n = 97 resident physicians	Open surgical skills: surgical knots, basic suturing, instrument handling (novice level)	Mixed simulation training (6x 1.5 hour weekly didactic teaching sessions, self- regulated home training with home training kit)	OSATS GRS scoring, blinded evaluators	No significant group differences in OSATS GRS scoring. Dyad group improved by 7.23 points, versus single group 6.94 (p = 0.881).
Abbott *et al.*, 2021 ^2^	United States of America	RCT	24/17, total n = 41 medical students	Symptomatic bradycardia simulation on day 1 (learning) and 4 (post-intervention)	Video debriefing after the day 1 simulation scenario	CAR, STAI, and cognitive load (paas) scales by students, time metrics by unblinded evaluators pre and post simulation on day 1 and 4	Significant group difference in anxiety and stress but non- significant group differences in cognitive load day 1 pre and post intervention. Non-significant group differences in anxiety, stress, cognitive load post day 4.

Abbreviations: CAR: Cognitive Appraisal Ratio; GRS: Global rating scale; MOCARS: modified coronary angiography rating scale; OSAUS: Objective Structured Assessment of Ultrasound Skills; OSATS: Objective Structured Assessment of Technical Skills; RIME: Reporter-Interpreter-Manager-Educator; STAI: State-Trait Anxiety Inventory

The mean MERSQI of included studies was 15 out of 18, and the range was 14.5 to 15.5. A detailed breakdown of the scoring of these studies is available as additional data (see the *Extended data* ^7^). Given the narrow range and mean score all studies were appraised to be of reasonable methodological quality.

The outcomes of each study are conceptualized with the Kirkpatrick model and are synthesized in Table 2.

**Table 2.** Overview of findings of studies that evaluated dyad and conventional learning outcomes in medical simulation as conceptualized with the Kirkpatrick model.

Studies that reached Kirkpatrick level 1 – Dyad experience (Learners’ reaction)						
Authors	Räder, 2014 ^13^	Shanks, 2013 ^18^	Abbott, 2021 ^2^	Tolsgaard, 2013 ^14^	Zetner, 2021 ^16^	
**Results**	Themes from qualitative interviewing: Reduced cognitive load, observational learning, communication, social aspects, meta-cognition	11-point Likert scale: Group difference in self-reported confidence after post-learning test below equivalence window (F _1,43_ = 2.03, p = 0.16). Single pre-test confidence: 4.2 (SD 2.09) Single post-test confidence: 7.10 (SD 1.37) Dyad pre-test confidence: 2.88 (SD 1.81) Dyad post-test confidence: 7.12 (SD 1.86)	Significant group difference in anxiety and stress day 1 pre and post intervention. Single (anxiety): 19.6 (SD 15.8). Dyad (anxiety): 7.6 (SD 14.4), max score 80. Single (stress): 1.8 (SD 1.8), Dyad (stress) 0.9 (SD 1.2). Non- significant group differences in anxiety, stress, and cognitive load post day 4. 58% of participants randomized to dyad group preferred the dyad dynamic. 77% of single participants preferred to train as an individual.	Likert-scale survey, (confidence rating): Single: 6.5 (SD 1.1), Dyad: 7.6, (SD 0.9), p < 0.001, effect size 1.16	Personal outcome rating: 5-point Likert-scale survey (mean) Single: 4.84. Dyads: 4.54. Qualitative results: dyads more motivated, had more fun, received beneficial feedback. Dynamic limited by challenges in coordinating schedules.	
Studies that reached Kirkpatrick level 2 – Knowledge acquisition (learning progression)						
Authors	Bjerrum, 2014 ^12^	Kowalewski, 2019 ^17^	Zetner, 2021 ^16^	Tolsgaard, 2015 ^15^	Shanks, 2013 ^18^	Abbott *et al.*, 2021 ^2^
**Results**	Pre-test to post-test: Significant main effect of test for all measures (p < 0.001). Learning (pre-test to retention test): No significant interaction between group and test for segments/min (p = 0.49), segments entered (p = 0.18), procedure time (p = 0.64), collisions (p = 0.46), red- out (p = 0.91), therefore parallel learning curves.	Conventional learning plateaued at attempt 5/7 for VR PT and 4/7 for VR LC. Dyad learning plateaued at attempt 4/7 for VR PT and VR LC. No significant difference between groups for operation time or number of attempts. Post-learning test, OSATS scores: Single, 40.2 (SD 9.8), Dyad 39.8 (SD 8.6), p = 0.995. Control (37.1 ± 7.4).	Time spent on self-regulated home training in minutes, median single 475 (IQR 360-475), dyads 395 (IQR 298-525). p = 0.668. Number of home training sessions median: single 12 (IQR 9-17), dyads 7 (IQR 5-10), p < 0.001. Pretest to posttest change median: single 7.0 (IQR 4.25 – 9), dyads 7.0 (IQR 6 – 9.5). p = 0.881.	Points per attempt during training: singles, 2.79, (SD 0.92), dyads, 5.88, (SD 1.13), p < 0.01. Therefore, dyad group had higher training efficiency. Immediate post-learning phase simulation test, OSAUS score: single 55.5, (SD 6.3), dyads 49.3, (SD 6.0).	No significant group difference in number of times supervisor consulted: Single 1.68, (SD 1.38), Dyad 1.96, (SD 1.86), no significant difference in total practice time (minutes): Single 20.94, (SD 6.2), Dyads 24.20, (SD 7.23). Immediate post-learning phase test, GRS scores: Single 3.31, (SD 0.68,), Dyad 3.52, (SD 0.68). Pre-test to post-test gains significantly greater for Dyad group compared to single group p = 0.02	4-day post- intervention evaluation. Single groups identified need for transcutaneous pacing more quickly (HR 2.26, p = 0.02) than dyads, but other outcomes were not statistically different; time to diagnose (HR 1.2, p = 0.63), call a rapid (HR 1.03, p = 0.94), acquire effective pacing (HR 1.5, p = 0.3).
Studies that reached Kirkpatrick level 3 – knowledge application (behavior)						
Authors	Bjerrum, 2014 ^12^		Räder, 2014 ^13^	Shanks, 2013 ^18^	Tolsgaard, 2013 ^14^	
**Results**	3-week post-teaching follow-up evaluation. Single: No significant drop in segments/min (p =0.27) or collisions (p = 0.20) versus Dyad: significant drop in segments/min (p = 0.002) and collisions performance (p < 0.001). However, group differences were not significant.		2-week post-teaching follow-up evaluation, MOCARS scores: Single 68, (SD 13), Dyad, 63, (SD 16), p = 0.18.	6-week post-teaching follow up evaluation, GRS scores: Single 3.21, (SD 0.79), Dyad 3.12, (SD 0.85), no significant difference p=0.69.	2-week post-teaching follow-up evaluation (simulated patient scenario, RIME based scoring), single: 36.9%, (SD 5.8%), dyads: 40.7%, (SD 6.6%), p = 0.04, effect size 0.61.	
Studies that reached Kirkpatrick level 4 – real world application (results)						
Authors	Tolsgaard, 2015 ^15^					
**Results**	1-day post-teaching follow-up live evaluation OSAUS score: single 48.4, (SD 11.8), dyads 56.3, (SD 14.8). Difference between groups 7.8%, (95% CI: -3.8-19.6%). More dyads, 72% achieved scores above pre-determined pass/fail level compared to single groups, 30%, p < 0.05					

Abbreviations: GRS: Global rating scale; IQR: Interquartile range; MOCARS: Modified coronary angiography rating scale; RIME: Reporter-Interpreter-Manager-Educator OSATS: Objective Structured Assessment of Technical Skills; OSAUS: Objective Structured Assessment of Ultrasound Skills; VR LC: Virtual reality laparoscopic cholecystectomy; VR PT: Virtual reality peg transfer.

### Dyad experience (learners’ reaction)

Overall, five studies reported level 1 evaluations ^2, 13, 14, 16, 18^. A follow-up survey for one of the studies included in this review was published as a separate paper ^19^. The methods used to capture learners’ reactions included questionnaires with Likert scales ^2, 14, 16, 18^ and qualitative interviewing ^13, 16^. Of the feedback obtained from dyad learners, three of the four sets of results ^13, 14, 18^ were post short training programs, where respondents would have been part of the dyadic dynamic for a timeframe ranging between 0.5 to 4 hours. The remaining study was conducted as a 6-week long training program, where participants worked as dyads both at the simulation facility and at home ^16^. Qualitative themes derived from interviewing participants who were acutely dyadic included a reduction of cognitive load, advantages of observational learning, communication, social aspects, and meta-cognition. These momentary dyads generally rated themselves as feeling more self-confident with independently applying the learned skilled compared to single group counterparts. Dyads who participated in the longer, 6-week study reported a high mean personal outcome rating 4.54/5, though this was inferior to that of the control group 4.84/5. Qualitative interviews with dyads yielded themes of higher motivation, levels of ‘fun’, and opportunities to receive direct and constructive feedback that would not otherwise have been attained. The prevailing limitation was challenges in synchronizing spare time between schedules.

### Knowledge acquisition (learning progression)

In total, six papers reported evaluations of learning progression ^2, 15– 18, 20^. The primary method of capturing this data was through conducting pre-training (baseline) tests and post-training test, while simulation device data and participant self-reporting were mainly used for secondary outcomes. Studies generally discovered equivocal learning progression and outcomes for dyads compared to singles. Tolsgaard and colleagues reported that the total ‘points’ per attempt with the simulation device was significantly higher for dyads, with a mean of 5.88 points compared to 2.79 points for single students, which could suggest more efficient training by the dyads ^15^. Another study found that improvement among dyads earlier at attempt 4 out of 7 for simulated peg-transfer, compared to 5 out of 7 for single groups ^17^. Further, Shanks and colleagues discovered that dyad pre-test to post-test changes were significantly higher than the single group ^18^. More recently in 2021, Zetner *et al.* reported that while there were no significant differences in total time spent for home training or pre-test to post-test changes between groups, the dyad groups only conducted on average, about seven home training sessions over 6-weeks, which was significantly lower than the 12 sessions the single group held ^16^. This again suggests that dyads were learning more efficiently than single learners. These findings contrast with that of Abbott *et al.*, who reported that single groups identified the need for transcutaneous pacing more rapidly in a post-test than singles after a single simulation scenario (HR 2.26, p = 0.02), although other measures such as time to diagnose bradycardia, or attain effective pacing were non-significant.

### Knowledge application (behavior)

Overall, four studies tested for knowledge retention and application, with the range of follow-up being from 2-weeks to 6-weeks since the final teaching session ^12– 14, 18^. One study used objective simulation data as the primary evaluation metric ^12^, whereas the other studies had blinded evaluators marking on standardized scoring systems. Bjerrum *et al.*, noted a statistically significant drop in performance by dyad groups in segments advanced by bronchoscope per minute and the number of collisions, whereas there was no statistically significant drop in performance by the single groups ^12^. However, the group differences for these results were not considered significant. Another study discovered that the dyads scored significantly higher than singles at 2-weeks follow-up, with a mean score of 40.7% versus 36.9% ^14^.

### Real world application (results)

Only one study evaluated for real world application of knowledge gained from simulation training ^15^. A live ultrasound was conducted independently by each participant the day following the conclusion of the training program, with performance assessed by a blinded evaluator. The authors discovered that dyad performance was non-inferior to the single groups, and that the live ultrasound performance scores were significantly greater than the pre-defined non-inferiority margin. However, significantly more dyads (72%) achieved scores above pre-determined pass/fail level compared to single groups (30%). 

## Discussion

This systematic review attempts to assess the efficacy of dyad learning versus single student learning under medical simulation conditions. Overall, dyad students reported positively regarding their experience in the dynamic, particularly in relation to the social and motivational aspects of it. Simulation workshops of one- or two-days length formed the bulk of studies analyzed. These workshops showed that dyadic simulation learning outcomes were non-inferior to single students. This non-inferiority appeared to be replicable in training programs of longer length and seemed to translate to a clinical context as well, suggesting that dyadic learning outcomes were not restricted to short programs or the simulation environment in which training occurred. 

Some studies did not report on student responses to dyad training, such as their mood, motivation, and boredom levels. Of those that did ^2, 13, 14, 16, 18^, the simulation workshops were of very short duration (a few hours). Certainly, one could predict positive feedback from students given that by the conclusion of the workshop the experience is likely still considered avant-garde and engaging. While ascertaining educational outcome is certainly an overarching objective of dyadic simulation training, retaining equal or better yet, accentuating student engagement is in line with contemporary efforts to diminish medical student dissatisfaction. Furthermore, there is substantial evidence that associates high student engagement with superior long term learning outcomes and teaching effectiveness ^21, 22^.

All but one of the studies implemented simulation-based training programs of workshop design with less than two days of teaching ^16^. Zetner and colleagues conducted the only study that evaluated dyadic efficacy in a training course of substantial length (6-weeks), and discovered no significant group differences in follow-up evaluation ^16^. The comparable learning outcomes between dyad groups and single student learners in short term (less than one day) training programs were preserved in this longer training program conducted by Zetner *et al.*, in that there were no significant group differences in follow-up evaluation. In all cases, the participants were starting from a beginner baseline.

Reduction of cognitive load has been cited as a chief benefit of dyad practice ^23^. Cognitive load theory is based on a conceptualization of human cognitive architecture as consisting of short-term working memory and long-term cognitive schemas ^24^. Working memory is limited in capacity and highly susceptible to decay, whereas cognitive schemas are highly automated and robust mental structures that enables the efficient extraction and organization of a specialized body of information ^25^. Learning has been described as the assembly of cognitive schemas, a process that inflicts cognitive load burden ^24^. One benefit of dyadic learning is that individuals share a collective working memory pool to co-construct a common mental schema ^23^. This enables the observing partner to diminish some of the burden, especially that of which is extraneous, which therefore allows the performing partner to consolidate on learning materials that are relevant for the construction of a schema. However, as mental schemas develop and cognitive processes become increasingly automated, the cognitive load burden is likely to decrease as participant skill level and exposure increases. This gives rise to arguably the most pressing issue that precludes a general recommendation in favor of dyad simulation – the issue of longitudinal outcome. The dyad groups could be performing at a level of non-inferiority or superiority in the short-term. However, if one were to model an entire curriculum based off the dyad model, such as a semester long clinical skills course, it is presently uncertain if this level of performance may endure.

Another potential shortcoming with the dyad model is a diminishment in the capacity for learners to be engaged in spaced, and repeated practical exposure to material over the long term. One could argue that if the results of these short-term studies were extrapolated, observational practice may account for this deficiency. Observational learning has been shown to augment the acquisition and retention of simple motor skills ^26, 27^. This property may be explained by mirror neurons located in the premotor cortex, inferior frontal gyrus, and inferior parietal lobe, which can be activated by either observing a second individual carrying out a motor act, or personally executing it ^28^. It is possible that the mirror neuron system may be more substantially activated in the setting of both executional and observational activity, rather than observation only for simple motor tasks ^29^. The dyad dynamic allows for both hands-on practice and the possibility of visualizing and assimilating movement sequences and adapting to the mistakes of their partner, which they may otherwise have not seen through personally. The product of this is therefore a sustained learning interface, whereby the time spent as an observer is not an idle discharge of time while one awaits their turn, but rather there is a constant development of skill. Observational learning between same-skill level participants may also naturally foster real-time exchange of dialogue, feedback, and supervision, which are all cognitively demanding. This correlates with existing theoretical groundwork, such as the proposition that cognitive processes play a prominent role during the foundational stages of motor skill acquisition ^30^. Further, timely feedback has been shown to not only transmit material information, but also promote motivation levels, which in turn enhances learning efficacy ^31^. Indeed, one study observing dyadic learning of a simple motor skill even concluded that learning advantages primarily stemmed from observation ^26^. However, theorized advantages of the dyad learning model, such as a reduction in individual cognitive load may be diluted with time and exposure. Further, physical training of motor skills results in distinct neurophysiologic changes that are more broadly referred to as consolidation ^32^. In contrast, while observational learning may also foster consolidation, it is of a different quality and likely has different neurobiological underpinnings. For instance, one study reported that if a variation of a learned motor skill were introduced, those who had the opportunity to practice it immediately performed it more accurately, whereas those who observed the new variation but then had an 8-hour delay before actively performing it themselves performed worse ^32^. Therefore, in a clinical simulation dynamic where learners may be barraged by demands for new motor inputs and are forced to be versatile in their history taking, physical exams, or practical interventions, the long-term strength in playing the observer role is uncertain.

An interesting point for the future is whether the dyadic dynamic may be effectively carried forward into non-simulation environments, such as clerkships. Presently, there is at least one primary paper reporting on dyads in non-simulation contexts ^33^. Certainly, clinical environments may be logistically difficult to evaluate, given that learning opportunities often present spontaneously. Nevertheless, given the improvement in confidence levels in being part of a dyadic dynamic, its potential utility may be worthwhile evaluating in certain situations such as the first-clerkship year or to help acclimatize foreign medical students on a global health rotation. If dyad non-inferiority is replicable under these circumstances, substantial improvements in teaching efficiency and expenditure may be achieved, which could potentially reduce the teaching burden of overloaded teaching hospitals. In addition, if the social and confidence benefits for dyadic medical students are retained, they would then be sustained over a more meaningful timeframe.

### Limitations

All interventions taught in a simulation environment were included for this review, whether it was purely clinical, such as simulated patient encounters, or highly practical and interventional, such as coronary angiography. Conducting an independent systematic analysis on each of the simulation skills taught to dyads and single groups would be the ideal. After all, the cerebral processes required for the acquisition of a pure motor skill are very different to that of undertaking a patient encounter. However, there is a paucity of original papers in the literature to allow for this, and consequently the focus and recommendations of this review are general. If the literature on any intervention expands in the future, this may allow for data pooling, and consequently a more specialized and authoritative meta-analysis on the topic.

Another limitation is the generally short duration of time that dyad learning was evaluated in the studies. As noted above, student engagement and subsequently learning may be related to the novelty of the dyad experimental process. Whether this is a confounding factor contributing to the non-inferiority of the dyad model has not been properly evaluated with longer term studies.

Cost-reduction in medical simulation education is a potential benefit of dyad teaching. However, as noted by commentators as early as 2015, the actual cost of implementing either dyad or conventional simulation education is not explicated by studies claiming such potentials ^34^. As of the time of writing, this issue prevails, with no study clarifying expenditures.

This systematic review chiefly represented the pedagogical aspect of dyadic teaching under simulation circumstances. While there were some inferences regarding possible translation of the partnership into clinical circumstances, the reality is that the clinical dynamic is a multifarious interplay of multiple stakeholders, such as physicians, nurses, and patients. Therefore, one cannot justify advancement of this dynamic into the clinical setting on academic grounds alone. Indeed, a recent study where 51 stakeholders were interviewed suggested that physicians and nurses may view a dyad as disruptive to the delicate clinical architecture, whereas students reported they were more engaged in clinical encounters, and patients were neutral to the presence of an additional student ^35^.

## Conclusions

The dyad method of instruction is increasingly being investigated for its efficacy across various aspects of medical education, such as in simulation contexts. The existing literature suggests that dyad practice at a novice level may enable a more efficient use of and allocation of simulation resources, without compromising on student learning. The dyad learning experience in courses of short duration is pleasant, with students highlighting the positive social and motivational aspects of it, though longer term studies are required to examine if this effect persists after the novelty has worn off. Further research on senior medical students or residents who have had some degree of exposure to an intervention is needed to discern whether these positive features of dyad training may permeate through to curriculums beyond that of a foundational level. Other avenues for research could include the longevity of the retention of skills as compared to traditional methods and comparison studies for the use of different sized groups, such as the effects of having three students or five students instead of two. Finally, studies that specify actual cost reductions associated with dyad teaching in simulation are needed to formalize the idea it is cost reductive.

## Data Availability

All data underlying the results are available as part of the article and no additional source data are required. *Extended data* Figshare: Dyad learning versus individual learning under medical simulation conditions: extended data and additional files. https://doi.org/10.6084/m9.figshare.20845126 ^7^. This project contains the following extended data: EXTENDED DATA.docx (2020 Prisma Flow chart, 2020 Prisma checklist, 2020 Prisma abstract checklist, and Medical Education Research Quality Instrument (MERSQI) scores of included studies.) Figshare: PRISMA checklist and flow diagram for ‘Dyad learning versus individual learning under medical simulation conditions: a systematic review’. https://doi.org/10.6084/m9.figshare.20845126 ^7^ Data are available under the terms of the Creative Commons Attribution 4.0 International license (CC-BY 4.0).
