# Supplementary material for: Dyad learning versus individual learning under medical simulation conditions: a systematic review
Source: MedEdPublish (2016). 2022 Nov 28;12:73. [Version 1] doi: 10.12688/mep.19285.1 (PMC10331849; doi:10.12688/mep.19285.1)
Supplement: Peer review history

# Peer review: Reviewer response for version 1

**DOI:** 10.21956/mep.20660.r33331

**Published:** 2023-06-12

**Authors:** Rod Moore

**Affiliations:** 1 Aarhus University, Aarhus, Denmark

Authors used PRISM criteria to make a systematic review that evaluated the efficacy of dyad learning in a medical simulation context.

 They identified papers that included eight studies from four countries that totaled 475 participants. Students reported positively on their experiences as dyads, especially regarding the social aspects of it. Studies showed non-inferior learning outcomes for dyads. There is some evidence to suggest that dyad learning outcomes may be replicable in a clinical context following simulation training. 

 The study design and results were well described and the conclusions followed the results very nicely. 

 I suggest that this manuscript be indexed as is.

Are the rationale for, and objectives of, the Systematic Review clearly stated?

Yes

Is the statistical analysis and its interpretation appropriate?

Yes

Are sufficient details of the methods and analysis provided to allow replication by others?

Yes

Are the conclusions drawn adequately supported by the results presented in the review?

Yes

Reviewer Expertise:

Dental education; dental student stress and coping

I confirm that I have read this submission and believe that I have an appropriate level of expertise to confirm that it is of an acceptable scientific standard.

# Peer review: Reviewer response for version 1

**DOI:** 10.21956/mep.20660.r32802

**Published:** 2022-12-21

**Authors:** Sören Huwendiek

**Affiliations:** 1 Institute for Medical Education, Department for Assessment and Evaluation, University of Bern, Bern, Switzerland

Thank you for this interesting read.

 Overall this is a well-designed and well-written systematic review on dyad vs. singular simulation training.

 I have only two minor comments:

1. In the following I cite a sentence from the discussion part (5 ^th^ paragraph):
  "Another potential shortcoming with the dyad model is a diminishment in the capacity for learners to be engaged in spaced, and repeated practical exposure to material over the long term."
  I think this is not fully self-evident. This should be explained and made explicit, especially also why “over the long term” (e.g. if two learners at the same time less exposure per learner; why long term related?)
2. On at least three occasions, I was a bit wondering that you put your results in the context of multiple “neurobiological mechanisms”, “neurobiological underpinnings” and “cerebral processes”.
  I would either go more deeply into this issue including citing relevant research or rather leave it fully out of the scope of this paper.. (I would recommend the latter).

Are the rationale for, and objectives of, the Systematic Review clearly stated?

Yes

Is the statistical analysis and its interpretation appropriate?

Yes

Are sufficient details of the methods and analysis provided to allow replication by others?

Yes

Are the conclusions drawn adequately supported by the results presented in the review?

Yes

Reviewer Expertise:

Virtual Patients, blended learning, formative/summative/programmatic/workplace-based assessment, qualitative research

I confirm that I have read this submission and believe that I have an appropriate level of expertise to confirm that it is of an acceptable scientific standard.
